# Technical note: A modified gamma evaluation method for dose distribution comparisons

**DOI:** 10.1002/acm2.12606

**Published:** 2019-07-07

**Authors:** Liting Yu, Tanya Kairn, Jamie Trapp, Scott B. Crowe

**Affiliations:** ^1^ Royal Brisbane & Women's Hospital Herston QLD Australia; ^2^ Queensland University of Technology Brisbane QLD Australia

**Keywords:** dose distribution comparison, gamma evaluation, patient specific quality assurance

## Abstract

**Purpose:**

In this work we have developed a novel method of dose distribution comparison, the inverse gamma (IG) evaluation, by modifying the commonly used gamma evaluation method.

**Methods:**

The IG evaluation calculates the gamma criteria (dose difference criterion, ΔD, or distance‐to‐agreement criterion, Δd) that are needed to achieve a predefined pass rate or gamma agreement index (GAI). In‐house code for evaluating IG with a fixed ΔD of 3% was developed using Python (v3.5.2) and investigated using treatment plans and measurement data from 25 retrospective patient specific quality assurance tests (53 individual arcs).

**Results:**

It was found that when the desired GAI was set to 95%, approximately three quarters of the arcs tested were able to achieve Δd within 1 mm (mean Δd: 0.7 ± 0.5 mm). The mean Δd required in order for all points to pass the gamma evaluation (i.e., GAI = 100%) was 4.5 ± 3.1 mm. The possibility of evaluating IG by fixing the Δd or ΔD/Δd, instead of fixing the ΔD at 3%, was also investigated.

**Conclusion:**

The IG method and its indices have the potential to be implemented clinically to quantify the minimum dose and distance criteria based on a specified GAI. This method provides additional information to augment standard gamma evaluation results during patient specific quality assurance testing of individual treatment plans. The IG method also has the potential to be used in retrospective audits to determine an appropriate set of local gamma criteria and action levels based on a cohort of patient specific quality assurance plans.

## 1 INTRODUCTION

Contemporary radiation therapy techniques can involve the use of complex radiation fields delivered with moving or static gantries to deliver modulated dose distributions. These techniques, which include intensity modulated radiation therapy (IMRT), volumetric modulated arc therapy (VMAT) and helical tomotherapy (HT), require careful quality assurance tests, to ensure that the planned dose distributions can be delivered by the treatment system^1^, ^2^, ^3^, ^4^, ^5^. Patient specific quality assurance (PSQA) is often performed prior to the radiation treatment, where the treatment beams are delivered to a phantom and the radiation doses are verified against those predicted in the treatment planning system (TPS). Dose comparisons are used to compare the evaluated dose distribution from measurements against the reference dose distribution from TPS calculations. Currently the gamma index method^6^ is the predominant dose comparison method being used clinically, although several alternative methods have been proposed^7^, ^8^, ^9^, ^10^, ^11^, ^12^.

Derived from the dose difference (ΔD) test and the distance‐to‐agreement (Δd) test^10^, the gamma index method calculates the difference between two dose grids in a combined spatial‐dose domain^6^. The result of the gamma test can be summarized by a single percentage value, usually referred to as the “pass rate” or “gamma agreement index” (GAI), which describes the percentage of points in the two dose distributions that agree within specified ΔD and Δd (producing a gamma value less than or equal to 1.0). Gamma evaluation results can also be plotted as a two‐dimensional gamma distribution with desired spatial resolution, as well as histograms, so that the locations of regions of disagreement can be identified and investigated^6^. The gamma evaluation method has the advantage of producing a quantitative measure based on both dose and spatial criteria, so that large dose differences occurring in high‐dose‐gradient regions do not disproportionately affect the results of the comparison. The gamma evaluation method has, however, been criticized for being less clinically intuitive than more conventional dose‐comparison methods^8^, being sensitive to dose grid resolution^13^ and having poor sensitivity and specificity to clinical dosimetric inaccuracies (when evaluated in terms of global dose difference)^14^, ^15^, ^16^, ^17^, ^18^, ^19^, ^20^.

Several alternative dose comparison methods have been proposed, to avoid some of the perceived weaknesses of the gamma index method^7^, ^8^, ^9^, ^10^, ^11^, ^12^. Specifically, several algorithms have been proposed which attempt to account for the differing levels of biological relevance associated with comparison results in different regions of the dose distribution. For example, the normalized agreement test (NAT)^7^, maximum allowed dose difference (MADD) method^8^ and the divide and conquer (D&C) gamma method^11^ all vary tolerances in dose‐differences in high‐dose or high‐dose‐gradient regions that may correspond to clinically important regions. A concept of radiobiological gamma index (Sumida method)^12^ has been proposed to integrate radiobiological parameters such as tumor control and normal tissue complication probabilities into gamma index calculation and produces more clinically relevant results. The proposed DVH‐based analysis^15^, ^21^, ^22^, ^23^, ^24^ allows different criteria to be used in each volume depending on clinical significance or required precision, which could be more relevant than simply judging the overall agreement^25^. These and other alternative dose comparison methods share the disadvantage of providing results that are difficult to compare and benchmark against historical data or other sources (other radiation oncology centres^26^, auditing bodies^27^ or established quality assurance guidelines^28^, ^29^), given the widespread adoption and acceptance of the gamma evaluation method.

This study investigates a modified gamma evaluation method, the “inverse gamma” (IG) evaluation, which calculates the gamma evaluation criteria (ΔD or Δd) that would be needed to achieve a predefined GAI. Li et al.^30^ proposed a similar approach where the passing percentage is fixed and combination of ΔD and Δd was calculated; however, has not been implemented into clinical QA. It is expected that the modified IG method proposed in this study will provide additional information for clinical PSQA, to augment standard gamma evaluation results by providing users with an indication of the minimum Δd for which a specified GAI can be achieved, when (for example) the ΔD is set to 3%.

## 2 MATERIALS AND METHODS

### 2.1 Standard gamma index method

The gamma index at a point *r*_r_ is defined as^6^:

(1) $$\gamma \left(r_{r}\right)=\min_{\left\{r_{e}\right\}}\left\{\Gamma \left(r_{e},r_{r}\right)\right\}$$

where

(2) $$\Gamma \left(r_{e},r_{r}\right)=\sqrt{\frac{\delta^{2}\left(r_{e},r_{r}\right)}{\Delta D^{2}}+\frac{r^{2}\left(r_{e},r_{r}\right)}{\Delta d^{2}}}$$

where δ(*r*_e_, *r*_r_) is the dose difference between evaluated and reference doses at point r, ΔD is the dose difference criterion, r(*r*_e_, *r*_r_) is the spatial distance between evaluated and reference dose points, and Δd is the distance‐to‐agreement criterion. The GAI is calculated as the percentage of points for which eq. (2) results in a gamma value less than or equal to 1.0, indicating agreement within the specified ΔD and Δd. The gamma index method implemented in this study used the global gamma normalization where the ΔD is normalized to the global maximum dose.

### 2.2 Inverse gamma with fixed ΔD (IG_ΔD_)

IG_ΔD_ calculates the minimum distance‐to‐agreement criterion (Δd) that is needed to achieve a specified GAI, given a fixed value of the dose difference criterion (ΔD). The fixed ΔD used in this study was selected to be 3%, denoted as IG_ΔD = 3%_. The IG algorithm performs iterative global gamma calculations with Δd increasing from 0 mm in 0.1 mm increments, until the specified GAI is reached and the required minimum Δd is reported. The resulting Δd can be denoted as Δd_GAI = 100% or 95%, ΔD = 3%_. Clinically this value would then be used to compare against a tolerance. The time required to perform the IG calculations is dependent on the number of iterations required. Lower Δd values require less time to calculate than higher Δd values. On average it takes a few minutes to run on a desktop PC, which is practical in clinical settings.

As an example, the ΔD was fixed at 3% in this work and the values of Δd required to achieve GAI values of 95% and 100% were investigated, for a pre‐existing set of VMAT PSQA results. The ΔD of 3% was chosen for this work because it is very widely recommended and used. Recent surveys^31^, ^32^ suggested that 3%/3 mm are currently the most commonly used gamma evaluation criteria, and the AAPM's task group report on IMRT commissioning (TG‐119)^28^ used 3%/3 mm and the AAPM's more recent task group report on modulated radiotherapy quality assurance (TG‐218)^29^ recommended 3%/2 mm. Investigations of cranial and extra‐cranial stereotactic radiotherapy have reported the use of 3%/2 mm, 3%/1.5 mm, 3%/1 mm and 3%/0.3 mm criteria^33^, ^34^, ^35^, ^36^, ^37^, ^38^, ^39^, ^40^.

### 2.3 Application

In‐house gamma evaluation code was developed using Python v 3.5.2, following the method proposed by Low et al.^6^. The code was validated by establishing agreement with the commercial SNC Patient software package within ± 1.5% [mean 0.3% difference], which falls within the range of variations due to minor differences in algorithm implementation^29^ between commercial gamma evaluation software packages reported by TG‐218. The in‐house code was also validated against the square‐field evaluation as per Low & Dempsey^6^.

The code was then modified to include the IG algorithm. A set of pre‐existing PSQA results, consisting of 53 arcs from 25 VMAT treatment plans measured using the ArcCheck (Sun Nuclear Corporation, Melbourne, USA) helical diode arrays, were arbitrarily selected and used to validate the performance of the code, by attempting to duplicate the gamma evaluation results produced during conventional PSQA tests using the SNC patient software (version 6.2.2) with Van Dyk global gamma analysis^41^ and 2D distance‐to‐agreement. The lower dose threshold (LDT) was set to 5%, which is consistent with the LDT used in the gamma evaluation.

The VMAT PSQA plans were delivered on Varian IX Clinac linacs (Varian Medical Systems, Palo Alto, USA) and measured using the ArcCheck model 1220, which is a cylindrical water‐equivalent phantom with 1386 diode detectors arranged in a spiral pattern. The detector array has its diameter and length of 21 cm. Measurements were taken with a solid PMMA insert placed in the ArcCheck's central cavity. The device was consistently set up so that the centre of the cylinder was at the isocentre. Comparisons were performed between the ArcCheck measured doses and the Eclipse (Varian Medical Systems, Palo Alto, California) calculated doses extracted by the SNC Patient software.

The performance of the standard gamma evaluation calculations by the in‐house code was verified by the Low and Dempsey method^6^ and established agreement with the output produced by the SNC Patient software. The in‐house code was then used to calculate IG_ΔD = 3%_ for all 53 measurements. When calculating IG_ΔD = 3%_, the target GAI was first defined as 100%, which represents the extreme situation where all points must pass the gamma test. The IG_ΔD = 3%_ analysis was then repeated, with the target GAI set to 95%, which corresponds to two standard deviations and is the action level most commonly used^31^.

## 3 RESULTS

The mean and standard deviation (SD) of IG_ΔD = 3%_ (when GAI was set to 100% and 95%), compared with their original gamma values, of the 53 VMAT arcs were calculated and displayed in Table 1. The detailed values for each individual arc were displayed in Table A1 in the Appendix.

**Table 1:** Mean and standard deviation of gamma and IG_ΔD = 3%_ at GAI 100% and 95% calculated from 25 previous VMAT PSQA data (53 arcs).

53 VMAT Arcs	Gamma pass rate (%) 2%, 2 mm, 5% LDT	Δd_GAI = 100%, ΔD = 3%_ (mm)	Δd_GAI = 95%, ΔD = 3%_ (mm)
Mean ± SD	97.9 ± 1.5	4.5 ± 3.1	0.7 ± 0.5

**Figure 1:**
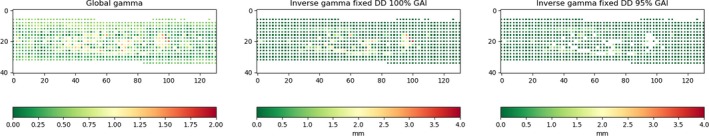
Global gamma distribution map (left). IG_ΔD = 3% _(GAI = 100%) Δd distribution map (middle). IG_ΔD = 3% _(GAI = 95%) Δd distribution map (right).

Figure 1 illustrates an example (arc 1) of the 2D global gamma distribution versus Δd distribution using IG_ΔD = 3% _with GAI 100% and 95%. Regions of high and low geometric uncertainties can be easily identified, which is not easily achievable by performing multiple gamma evaluation with varying Δd criteria.

**Figure 2:**
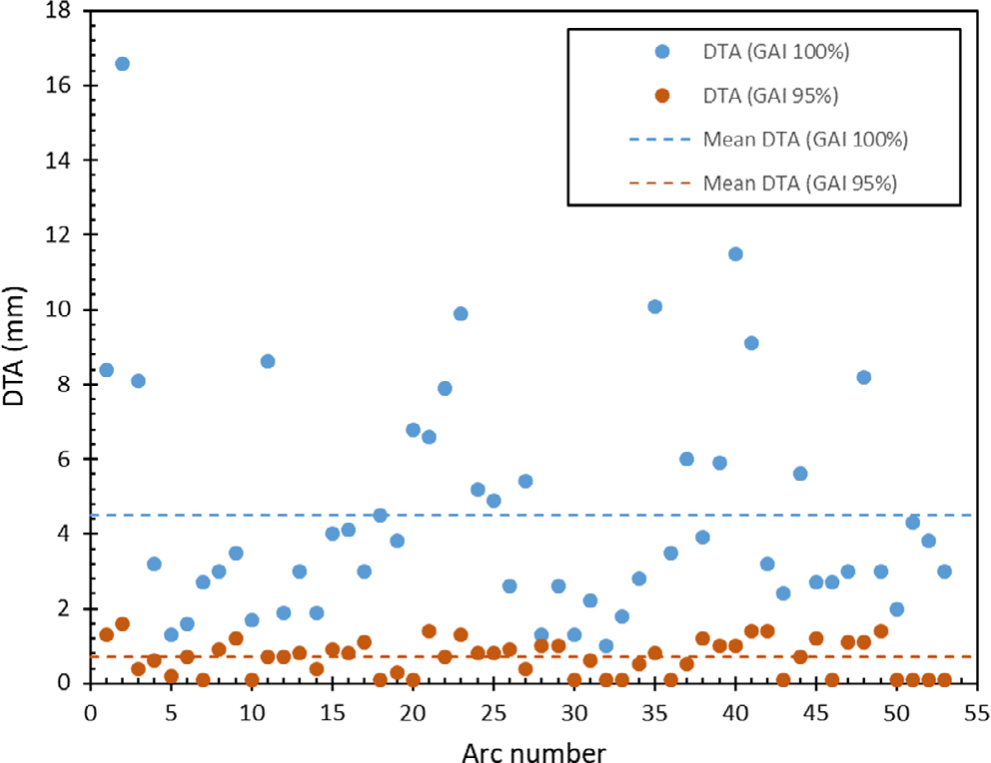
DTA values (Δd) required to achieve GAI values of 95% (darker/orange plot symbols) and 100% (lighter/blue plot symbols), with a set ΔD of 3%.

Figure 2 illustrates the results of the IG analysis of the VMAT PSQA results, showing the Δd required to achieve the specified GAI values for each of the 53 arcs, when the ΔD is fixed at 3%. The mean Δd required to achieve a GAI of 100% was 4.5 ± 3.1 mm. The number of arcs that achieved a GAI of 100% only when Δd was 10 mm or more provides a graphic indication of the clinical unsuitability of requiring that all points pass the gamma evaluation. The mean Δd required to achieve the more‐conventional GAI of 95% was 0.7 ± 0.5 mm, with the majority arcs (75.5%) requiring Δd less than 1 mm.

Examination of the results in Fig. 2 indicates that if initial PSQA testing of these arcs had used gamma evaluation criteria of 3%/2 mm, all arcs would have achieved a GAI greater than 95%. Data in Fig. 2 also indicate that that if initial PSQA testing of these arcs had used gamma evaluation criteria of 3%/1 mm, then 75.5% of the arcs would have achieved a GAI greater than 95%.

The highest Δd calculated is from arc 2 using GAI of 100%, which indicated that a Δd of nearly 17 mm is needed for all points to pass gamma when the ΔD value is set to 3%. However, when GAI of 95% is used, Δd has been significantly dropped to only 1.6 mm. This behavior will be discussed in the next section.

## 4 DISCUSSION

Standard gamma evaluation method cannot provide definitive results (whether in the form of a single numeric GAI value or a 2D gamma map) to answer questions about the magnitude or source of error (whether because of the disagreement in ΔD or Δd). The AAPM's task group report on PSQA (TG‐218)^29^ has recently recommended that reports of PSQA results include the percentage of gamma values greater than 1.5, as a means to provide an indication of the magnitude of disagreement, but the gamma value of 1.5 is also difficult to interpret – it may be caused by a difference in dose or position or both. DVH based analysis^15^, ^21^, ^22^, ^23^, ^24^ has potential possibilities to evaluate the clinical importance of any observed differences by seeing the effect on specific structures, which could be more relevant than simply judging the overall agreement^25^. The IG method proposed in this study provides a means to generate two‐dimensional maps of ΔD or Δd results (in the same way that two‐dimensional maps of gamma values are currently provided by commercial gamma evaluation software packages), so that regions of high and low dosimetric or geometric uncertainty can be easily identified. Now with the ability of some commercial software reconstructing 2D dose map onto 3D CT dataset, potentially the shifts identified here might allow correlating the identified geometrical shifts with the borders of organs at risk (OAR) and planning target volumes (PTVs). This method may help address the challenge of implementing DVH or anatomical‐structure‐based analysis methods into the clinical workflow^25^. For example, the IG method could potentially allow the Δd results identified at the periphery of target structures to be evaluated for compliance with stereotactic criteria, while the Δd throughout the rest of the dose distribution are evaluated more leniently.

TG‐218 also states that the Δd should be a function of the clinical necessity of placing steep dose gradients, yet discovering the locations of the dose gradient errors in the patient is difficult with the commonly used IMRT QA methods^29^. The IG method proposed in this study will tackle this difficulty by searching for a single Δd for the structures that provides a pass without running multiple gamma evaluations for each structure. This method also allows a simple check of the value against a tolerance table.

This IG method has been proposed as a means to provide additional information, to augment standard gamma evaluation results during PSQA testing of individual treatment plans. For example, if the local PSQA action threshold is a GAI of 95%, and a given treatment plan achieves a GAI of 94%, using criteria of 3%/2 mm, instead of iteratively changing the Δd criterion by manual trial and error, the proposed IG_ΔD = 3% _method can be used as an additional test of verification more efficiently. This method can be used to identify the minimum Δd for which a GAI of 95% is achievable for that plan. The two‐dimensional Δd distribution map can also identify the region of high and low Δd values. In cases where it is concerned that there are points having deviations larger than the selected gamma criteria, however, are not able to be identified due to using GAI of 95%, the GAI of 100% can be used to eradicate this common weakness of the conventional gamma evaluation method. It is worth mentioning that the weakness of using GAI of 100% is that it is very sensitive to noise. In cases where there are problems with even a single pixel (noise, dead pixel, etc), the IG method could potentially keep doing iterations until reaching the maximum specified gamma criterion, which is both time consuming and giving less meaningful results (for example arc 2). Therefore, care needs to be taken when using GAI of 100%. The GAI values of near 100%, for example 99%, 99.5%, are highly recommended in order to avoid the disturbance of random noise in the dataset.

Similarly, the IG method may be used to retrospectively audit cohorts of PSQA results (producing results similar to Fig. 2), so that the suitability of existing or proposed gamma evaluation criteria and action thresholds can be evaluated. The data produced by applying IG analysis to aggregated PSQA results may be evaluated using statistical process control (SPC) methods, such as those have been used to identify uncontrolled behaviors in linac and tomotherapy quality assurance results^42^, ^43^, ^44^, ^45^.

The 3% ΔD criterion was selected in this study because it is widely used, in combination with a range of Δd values^28^, ^29^, ^31^, ^33^, ^35^, ^36^, ^37^, ^38^, ^39^, ^40^, but the use of 3% as a ΔD criterion is not universal^26^, ^34^. Similarly, GAI values of 100% and 95% were selected as examples of extreme and commonly‐used PSQA action levels, respectively. The IG method can, however, be used with any other ΔD or GAI values, to fit the needs of individual centres.

The IG method also offers more variability and flexibility than simply fixing the ΔD criterion. The algorithm is amenable to using a fixed Δd to identify the ΔD that would produce a specified GAI. Alternatively, the ratio of ΔD to Δd can be fixed, enabling the IG algorithm to identify the pair of criteria (related to each other via a set ratio) that produce a specified GAI. (Examples of the use of these two additional forms of the IG index are provided in the appendix.).

For centres that are committed to using the standard gamma evaluation method for PSQA, the IG indices developed in this study could be used to investigate or justify the choice of gamma criteria for ongoing PSQA use, based on combination of the local treatment technique and measuring device.

## 5 CONCLUSIONS

A novel dose comparison method called the inverse gamma (IG) method has been developed. The IG_ΔD = 3%_ index has been tested on 25 retrospective VMAT PSQA plans (53 arcs). This index was proven useful to quantify the minimum Δd based on given ΔD of 3% in order to pass a given GAI. This method has the potential to be implemented clinically to perform additional analysis of failed plans and to provide those who prescribe, plan and test modulated radiotherapy treatments with more detailed dosimetric information about the reliability with which planned doses can be delivered. The IG method also has the potential to be used in retrospective internal and inter‐departmental audits, to evaluate the suitability of the local gamma evaluation criteria.

## CONFLICT OF INTEREST

The authors have no relevant conflict of interest to disclose.

## Figures and Tables

**Figure A1:**
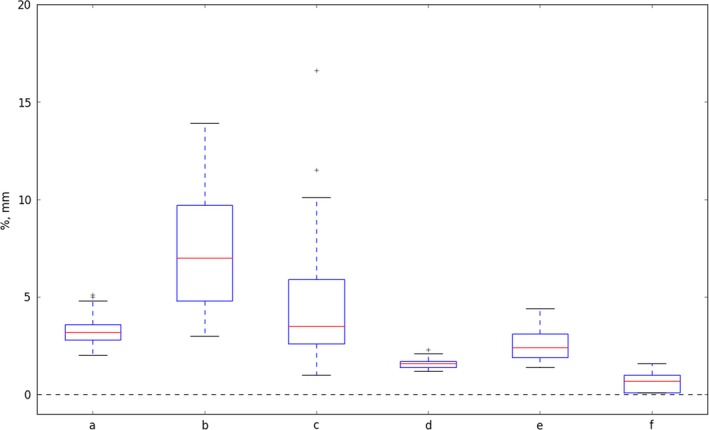
Distribution of IG_ΔD/Δd __= 1.0_, IG_Δd_ _= 1 mm _and IG_Δ__D_ _= 3%_ at GAI 100% and 95% of 53 individual arcs, including mean and standard deviation. From left to right are a) IG_ΔD/Δd __= 1.0_ at 100%, b) IG_Δd_ _= 1 mm _at 100%, c) IG_ΔD_ _= 3%_ at 100%, d) IG_ΔD/Δd __= 1.0_ at 95%, e) IG_Δd_ _= 1 mm _at 95% and f) IG_ΔD = 3%_ at 95% respectively.

**Figure A2:**
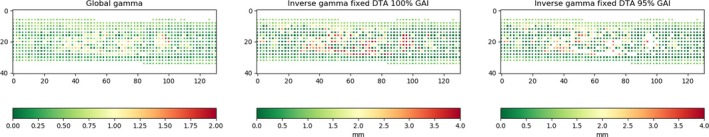
Global gamma distribution map (left). IG_Δd = 1mm _(GAI = 100%) Δd distribution map (middle). IG_Δd = 1mm _(GAI = 95%) Δd distribution map (right).

## APPENDIX 1

The body of this manuscript describes the IG method, using the example of keeping the ΔD fixed and identifying the minimum Δd required to achieve a specified GAI for each PSQA comparison (abbreviated to IG_ΔD_). Two additional forms of the IG algorithm have been investigated: inverse gamma with fixed Δd (IG_Δd_) and inverse gamma with fixed dose‐distance‐ratio (IG_ΔD/Δd_).

As an obvious analogue to IG_ΔD_, IG_Δd_ keeps the Δd fixed and calculates the minimum ΔD required to achieve a specified GAI for each PSQA comparison. The algorithm performs iterative global gamma calculations of ΔD from 0% with 0.1% increment until the minimum ΔD were found to achieve a GAI that is no less than the predefined threshold GAI value. To provide an indication of the results achievable using IG_Δd_, the 53 VMAT PSQA results used in this work were re‐evaluated using a fixed Δd of 1 mm, denoted as IG_Δd = 1mm_ (see Fig. A1). A comparable example of the resulting ΔD distribution when applying the IG_Δd = 1mm _method is illustrated in Fig. A2. As we can see that Fig. 1 is a Δd distribution map, in which the points with higher values are indication of regions where larger distance‐to‐agreement disagreement occurs. Likewise, Fig. A2 is a ΔD distribution map where the points with higher values indicate regions of larger dose difference disagreement. In comparison, neither Δd nor ΔD information is apparent on the gamma distribution map.

The IG_ΔD/Δd_ concept is based on the gamma index definition (global normalization). The difference is that instead of calculating GAI based on fixed ΔD and Δd in the gamma method, IG_ΔD/Δd_ calculates the minimum ΔD and Δd based on fixed GAI and dose‐difference ratio (ΔD/Δd). The dose‐difference ratio used in this study was unity (e.g., 3%, 3 mm, 2%/2 mm, 1%/1 mm, etc), denoted as IG_ΔD/Δd = 1.0_. While keeping a constant ratio of 1, the algorithm performs iterative global gamma calculations of GAI using ΔD and Δd ranging from 1%/ 1 mm with 0.1%/mm increment until the resulting GAI equals to or above the predefined threshold GAI value (see Fig. A1).

Figure A1 shows how the IG results can vary, depending on the selection of the GAI and on which specific IG method (fixed ΔD, fixed Δd or fixed ΔD/Δd) is used for the comparison. Clearly, the ΔD and Δd values required to achieve a “passing” result are higher when the GAI action threshold is 100%, compared to when the GAI action threshold is 95%. Similarly, the ΔD values required to exceed the GAI action threshold are higher when the Δd is set to 1 mm, than when the Δd is allowed to vary at a constant ratio with the ΔD.

Figure A1 also provides an indication of how the indices developed in this study can be used to evaluate locally used or proposed gamma criteria and GAI, as part of a statistical process control (SPC) process. In Fig. A1, the mean ΔD and Δd criteria that result from an IG_ΔD/Δd = 1.0_ evaluation of the VMAT PSQA results, with a GAI of 95%, is 1.6 ± 0.2%/1.6 ± 0.2 mm, which is just within 2%/2 mm. This suggests that if 2%/2 mm was used in a standard gamma calculation, the resulting mean GAI would be slightly higher than 95% (in fact, the results of this study show that it is 97.9 ± 1.5%). This result suggests that using the gamma criteria of 2%/2 mm and GAI of 95% is suitable for the cohort of plans. This finding is consistent with the results estimated using the SPC method^29^ for the same cohort of patients. Similarly, Fig. A1 suggests that if a center decided to use a GAI of 100%, then the suitable gamma criteria they should be using is 4%/4 mm, assuming a unity dose‐to‐distance ratio is preferred.

**Table A1:** Results of IG_ΔD/Δd = 1.0_, IG_Δd = 1mm_ and IG_ΔD = 3%_ calculated for 53 VMAT arcs using 100% GAI and 95% GAI respectively.

Patient	arc	Plan	100% pass rate			95% pass rate		
			%, mm	1mm DTA	3%, mm	%, mm	1mm DTA	3%, mm
1	1	Abdomen	4.3	11.0%	8.4	1.9	3.5%	1.3
	2		3.9	10.5%	16.6	2	3.8%	1.6
2	3	Lung	4	4.4%	8.1	1.5	2.3%	0.4
	4		3.1	7.9%	3.2	1.7	2.6%	0.6
3	5	Brain	2.5	3.4%	1.3	1.4	1.7%	0.2
	6		2.1	4.4%	1.6	1.4	2.0%	0.7
	7		3	3.5%	2.7	1.4	1.7%	0.1
4	8	Boost RT BM	3	7.0%	3	1.4	2.4%	0.9
	9		3.3	13.9%	3.5	1.7	4.1%	1.2
5	10	RTLUNG	2.4	5.7%	1.7	1.5	1.9%	0.1
	11		3.2	5.9%	8.6	1.8	2.5%	0.7
6	12	LT BM BOOST	2.7	4.8%	1.9	1.3	1.8%	0.7
	13		3	8.8%	3	1.4	2.5%	0.8
7	14	Oesophagus	2.6	4.4%	1.9	1.6	2.1%	0.4
	15		3.2	7.7%	4	1.7	2.8%	0.9
8	16	BM Boost	3.3	7.4%	4.1	1.4	1.6%	0.8
	17		3	12.4%	3	1.6	3.2%	1.1
9	18	GOJ	3.5	6.1%	4.5	1.5	1.9%	0.1
	19		3.5	7.2%	3.8	1.7	2.6%	0.3
10	20	Pelvis	3.6	4.3%	6.8	1.8	2.3%	0.1
	21		4	6.0%	6.6	2.3	3.3%	1.4
11	22	RTHN	3.5	6.8%	7.9	1.6	2.4%	0.7
	23		4.7	9.8%	9.9	2	3.5%	1.3
12	24	Pelvis	3.4	8.0%	5.2	1.9	2.8%	0.8
	25		4.8	9.4%	4.9	2	2.9%	0.8
13	26	Pelvis boost	2.9	6.2%	2.6	1.6	2.6%	0.9
	27		4.2	9.3%	5.4	2.1	3.6%	0.4
14	28	Oesophagus	2	4.8%	1.3	1.3	1.7%	1
	29		2.7	11.1%	2.6	1.7	3.0%	1
15	30	LT CHEST	2.3	4.1%	1.3	1.3	1.5%	0.1
	31		2.6	6.2%	2.2	1.6	2.1%	0.6
16	32	Brain	2.3	3.0%	1	1.4	1.8%	0.1
	33		2.4	7.8%	1.8	1.6	2.2%	0.1
17	34	RTHN	3	5.7%	2.8	1.7	2.3%	0.5
	35		4.1	12.8%	10.1	1.6	2.4%	0.8
	36		3.2	4.3%	3.5	1.3	1.7%	0.1
18	37	Rforehead	5.1	7.9%	6	1.7	2.5%	0.5
	38		3.6	9.7%	3.9	2	3.2%	1.2
	39		5	6.6%	5.9	1.7	2.9%	1
19	40	RT BM boost	4.5	12.7%	11.5	1.6	2.4%	1
	41		4.4	13.3%	9.1	1.8	4.4%	1.4
20	42	LT BM BOOST	3.1	10.7%	3.2	1.8	3.8%	1.4
	43		2.7	6.6%	2.4	1.5	1.8%	0.1
21	44	LT BM BOOST	3.5	4.8%	5.6	1.5	2.5%	0.7
	45		2.8	10.3%	2.7	1.7	4.1%	1.2
22	46	RT BRAIN	2.9	6.9%	2.7	1.5	1.8%	0.1
	47		3	8.4%	3	1.6	3.1%	1.1
23	48	LT BM BOOST	3.6	11.0%	8.2	1.7	3.4%	1.1
	49		3	12.0%	3	1.9	4.1%	1.4
24	50	Gynae	2.7	3.9%	2	1.2	1.4%	0.1
	51		3.7	7.2%	4.3	1.5	2.1%	0.1
25	52	ABDO + Pelvis	3.4	5.1%	3.8	1.3	1.6%	0.1
	53		3	3.9%	3	1.4	1.6%	0.1
Mean			3.3	7.5%	4.5	1.6	2.6%	0.7
SD			0.7	2.9%	3.1	0.2	0.8%	0.5
